# Correction: Back to normal? The health care situation of home care receivers across Europe during the COVID-19 pandemic and its implications on health

**DOI:** 10.1371/journal.pone.0319693

**Published:** 2025-02-21

**Authors:** Michael Bergmann, Melanie Wagner

The Acknowledgment section is not indicated. The Acknowledgement section is as follows: This paper uses data from SHARE Waves 1, 2, 3, 4, 5, 6, 7, 8, and 9 (DOIs: 10.6103/SHARE.w1.900, 10.6103/SHARE.w2.900, 10.6103/SHARE.w3.900, 10.6103/SHARE.w4.900, 10.6103/SHARE.w5.900, 10.6103/SHARE.w6.900, 10.6103/SHARE.w7.900, 10.6103/SHARE.w8.900, 10.6103/SHARE.w8ca.900, 10.6103/SHARE.w9ca900) see Börsch-Supan et al. (2013) for methodological details. The SHARE data collection has been funded by the European Commission, DG RTD through FP5 (QLK6-CT-2001-00360), FP6 (SHARE-I3: RII-CT-2006-062193, COMPARE: CIT5-CT-2005-028857, SHARELIFE: CIT4-CT-2006-028812), FP7 (SHARE-PREP: GA N°211909, SHARE-LEAP: GA N°227822, SHARE M4: GA N°261982, DASISH: GA N°283646) and Horizon 2020 (SHARE-DEV3: GA N°676536, SHARE-COHESION: GA N°870628, SERISS: GA N°654221, SSHOC: GA N°823782, SHARE-COVID19: GA N°101015924) and by DG Employment, Social Affairs & Inclusion through VS 2015/0195, VS 2016/0135, VS 2018/0285, VS 2019/0332, VS 2020/0313 and SHARE-EUCOV: GA N°101052589 and EUCOVII: GA N°101102412. Additional funding from the German Ministry of Education and Research, the Max Planck Society for the Advancement of Science, the U.S. National Institute on Aging (U01_AG09740-13S2, P01_AG005842, P01_AG08291, P30_AG12815, R21_AG025169, Y1-AG-4553-01, IAG_BSR06-11, OGHA_04-064, BSR12-04, R01_AG052527-02, HHSN271201300071C, RAG052527A) and from various national funding sources is gratefully acknowledged (see www.share-eric.eu).
