# Supplementary material for: Back to normal? The health care situation of home care receivers across Europe during the COVID-19 pandemic and its implications on health
Source: PLoS One. 2023 Oct 23;18(10):e0287158. doi: 10.1371/journal.pone.0287158 (PMC10593209; doi:10.1371/journal.pone.0287158)
Supplement: S1 Table — Data: SHARE Wave 8 COVID-19 Survey 1 and SHARE Wave 9 COVID-19 Survey 2, Release version: 8.0.0 (n = 48,058, respectively; weighted) with 95% confidence intervals in brackets. (DOCX) [file pone.0287158.s001.docx]

**S1 Table: Prevalence of receiving home care during the pandemic (in %) by age and health groups**

|  | **SCS1 (2020)** | **SCS2 (2021)** |
| --- | --- | --- |
| Age (50-64 years) | 1.3 | 2.5 |
|  | [0.9; 1.8] | [1.8; 3.2] |
| N | 13,856 | 12,025 |
| Age (65-79 years) | 4.2 | 6.0 |
|  | [3.7; 4.6] | [5.5; 6.5] |
| N | 25,963 | 26,579 |
| Age (80+ years) | 19.1 | 25.7 |
|  | [17.6; 20.6] | [24.0; 27.3] |
| N | 8,209 | 9,401 |
| No limitations in ADL | 3.0 | 5.3 |
|  | [2.7; 3.3] | [4.8; 5.7] |
| N | 42,627 | 42,607 |
| 1+ limitations in ADL | 22.8 | 25.7 |
|  | [20.4; 25.3] | [21.0; 30.5] |
| N | 4,695 | 4,691 |

Data: SHARE Wave 8 COVID-19 Survey 1 and SHARE Wave 9 COVID-19 Survey 2, Release version: 8.0.0 (n=48,058, respectively; weighted) with 95% confidence intervals in brackets.
