# Supplementary figures and images for: Back to normal? The health care situation of home care receivers across Europe during the COVID-19 pandemic and its implications on health
Source: PLoS One. 2023 Oct 23;18(10):e0287158. doi: 10.1371/journal.pone.0287158 (PMC10593209; doi:10.1371/journal.pone.0287158)

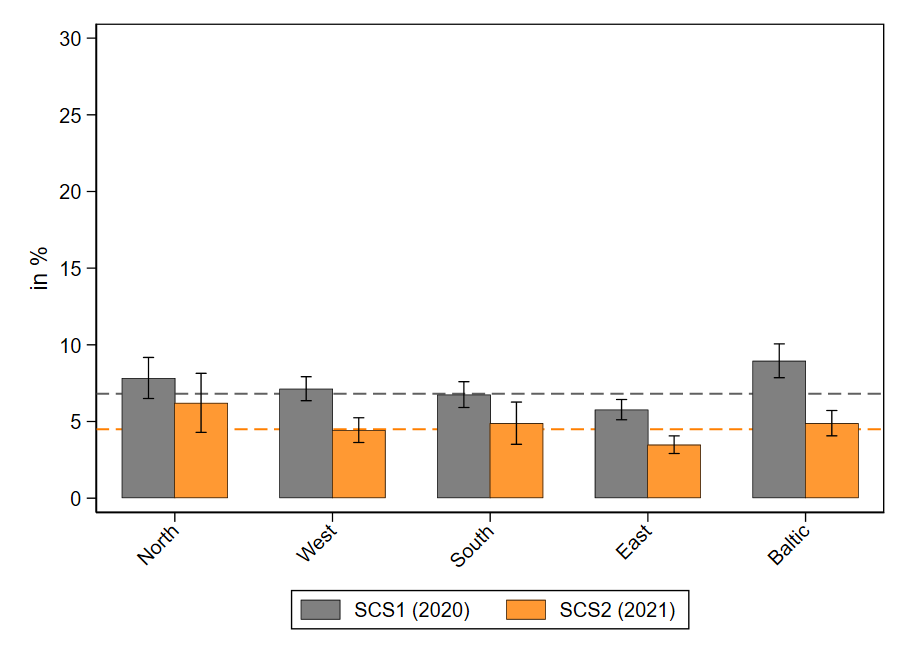

Supplement: S1 Fig — Data: SHARE Wave 8 COVID-19 Survey 1 and SHARE Wave 9 COVID-19 Survey 2, Release 8.0.0 (n = 48,016 and 47,958 respectively; weighted) with 95% confidence intervals. (TIF) [file pone.0287158.s002.tif]

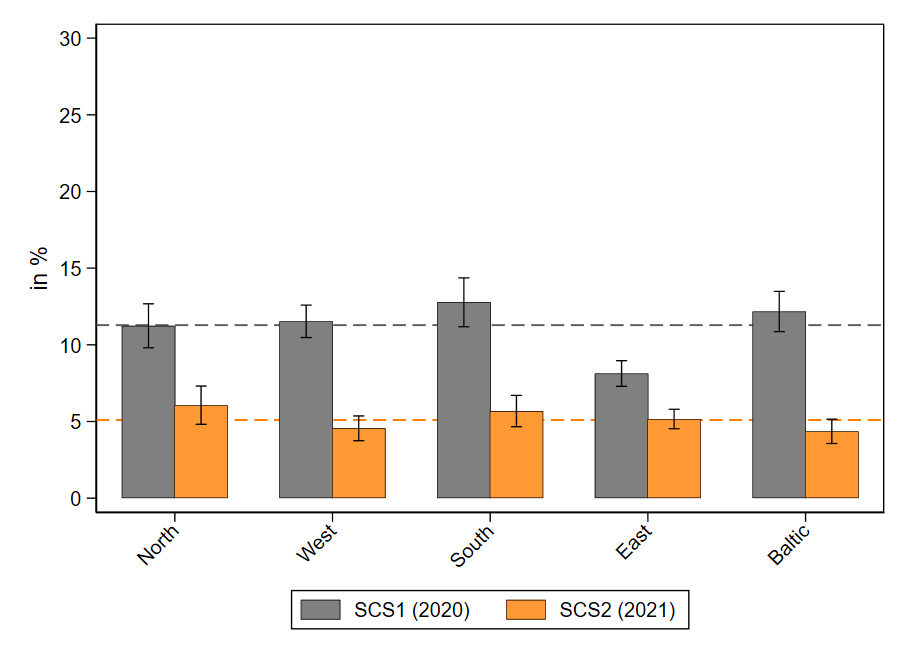

Supplement: S2 Fig — Data: SHARE Wave 8 COVID-19 Survey 1 and SHARE Wave 9 COVID-19 Survey 2, Release 8.0.0 (n = 48,012 and 47,921 respectively; weighted) with 95% confidence intervals. (TIF) [file pone.0287158.s003.tif]
